# Low-NaCl pretreatment promotes seedling growth of Chinese white radish under water deficit via osmotic and energy regulation

**DOI:** 10.1186/s12870-026-08175-9

**Published:** 2026-01-20

**Authors:** Nutkamol Masepan, Patcharapon Chaimung, Sitthisak Intarasit, Usawadee Chanasut, Jarunee Jungklang

**Affiliations:** 1 Department of Biology, Faculty of Science, Chiang Mai University, Chiang Mai, 50200 Thailand; 2 Ph.D.’s Degree Program in Biology (International Program), Faculty of Science, Chiang Mai University, 50200 Chiang Mai, Thailand

**Keywords:** Chinese white radish, NaCl pretreatment, Water deficit, Photosynthetic and respiratory activity, Osmotic regulation, Energy status

## Abstract

Pretreatment with sodium chloride (NaCl) has been reported to enhance plant tolerance to subsequent water deficit; however, the underlying energy-related mechanisms, particularly during early seedling development, remain insufficiently understood. This study aimed to (1) evaluate the effects of low NaCl and water-deficit conditions on growth, water status, chlorophyll and proline contents in Chinese white radish seedlings, and (2) investigate the influence of NaCl pretreatment on photosynthetic activity, respiratory CO₂ production, sugar accumulation, and cellular energy status under water-deficit condition. Seeds were germinated in sand culture and watered with 0, 10, or 20 mM NaCl for 4 days, followed by transferring the seedlings to 0, − 0.5, or − 1.0 MPa mannitol solutions for 3 days. Growth parameters, water status, chlorophyll and proline contents were assessed in 7-day-old seedlings. Low-NaCl pretreatment enhanced shoot height, fresh weight, yield, water content, degree of succulence and proline content under water deficit. The optimal conditions (10 mM NaCl and − 0.5 MPa) were subsequently used to evaluate Hill’s reaction activity, CO₂ production, sugar contents, and energy status. NaCl-pretreated seedlings exhibited increased CO₂ production; higher fructose, glucose, and sucrose contents; elevated ATP/ADP and ATP/AMP ratios; and a higher adenylate energy charge under water deficit. Overall, the findings demonstrate that 10 mM NaCl pretreatment enhances the growth of Chinese white radish seedlings under water-deficit condition by improving osmotic regulation and maintaining energy homeostasis.

**Supplementary Information:**

The online version contains supplementary material available at 10.1186/s12870-026-08175-9.

## Introduction

Water-deficit stress, or drought stress, is one of the most severe environmental stresses, particularly under current climate-change conditions. This stress arises from reductions in tissue water content, turgor pressure and water potential, which lead to wilting, stomatal closure and decreases in plant growth and productivity. Water-deficit stress affects plant growth and development at multiple levels, with cell enlargement generally being more sensitive to dehydration than cell division [1, 2]. Water-deficit stress triggers excessive production and accumulation of reactive oxygen species (ROS) in plant cells including superoxide radicals (O₂•⁻) and hydroxyl radicals (•OH), which damage essential macromolecules such as proteins, membrane lipids and nucleic acids, thereby disrupting key physiological and biochemical processes of photosynthesis and cellular respiration [3].

Moreover, water-deficit stress impairs electron transport in both chloroplasts and mitochondria, thereby reducing adenosine triphosphate (ATP) synthesis. ATP is widely recognised as the primary energy currency of the cells. It is synthesised through the phosphorylation of adenosine diphosphate (ADP) and inorganic phosphate (Pi) during photophosphorylation and oxidative phosphorylation. The ATP/ADP ratio serves as a key indicator of cellular energy status. Previous studies have reported that water-deficit stress decreases ATP production and disrupts ATP homeostasis in plant cells, leading to the reduction of cellular energy [4, 5]. Under energy-deficit conditions, adenosine monophosphate (AMP) levels increase while ATP levels decline due to consumption, resulting in a substantial reduction in the ATP/AMP ratio, which is widely regarded as a sensitive indicator of cellular energy deficiency [6–8].

Low concentrations of NaCl (5–50 mM) have been reported to promote the growth and development of several plant species, particularly halophytes and plants exposed to abiotic stress [9, 10]. Low accumulation of sodium (Na⁺) and chloride (Cl⁻) ions contributes to osmotic adjustment by enhancing soluble sugars and proline levels [11]. Several studies have shown that low-NaCl pretreatment enhances plant tolerance to water-deficit conditon through mechanisms such as improved osmotic adjustment, maintenance of endogenous hormone homeostasis, reinforcement of antioxidant defence systems and enhanced photosynthetic performance in species including *Atriplex halimus*, white clover and cotton [12–14].

Chinese white radish, a halophytic species, tolerates high salinity (0.61–1.2 dS/m EC 1:5; 0.4–0.8% soil salt) [15–17], while young seedlings (Kaiware) exhibit increased fresh weight when exposed to low NaCl (10 mM) [9] and can grow under 0.4–0.8 dS/m salinity in sand culture [18]. These seedlings are rich in vitamins (B-group and C), minerals (Cl, K, Zn, Fe, Mg, Mn and P) and bioactive compounds such as polyphenols and glucosinolates [19, 20], making them a nutritionally valuable food source. Our previous study demonstrated that 10 mM NaCl enhanced the growth of 4-day-old seedlings by increasing water content and upregulating aquaporin genes in the cotyledons [21].

Research on the growth and physiological responses of Chinese white radish seedlings under water-deficit conditions remains limited, with the influence of low-NaCl pretreatment on the energy status of seedlings subsequently exposed to water deficit not systematically examined. Therefore, this study (1) evaluated the effects of low NaCl and water-deficit conditions on growth, water status, chlorophyll and proline contents in Chinese white radish seedlings and (2) investigated the energy status and related physiological parameters of seedlings pretreated with low NaCl under water-deficit condition. Low concentration of NaCl pretreatment was hypothesised to enhance cellular water uptake and maintain energy homeostasis, thereby supporting seedling growth under water-deficit condition. The findings from this study enhance our understanding of the energy-related mechanisms by which low-NaCl pretreatment promotes the growth of Chinese white radish seedlings under water deficit and offer valuable insights for improving seedling performance under water deficit and other environmental stress conditions.

## Materials and methods

### Plant materials, establishment and experimental design

**Fig. 1:**
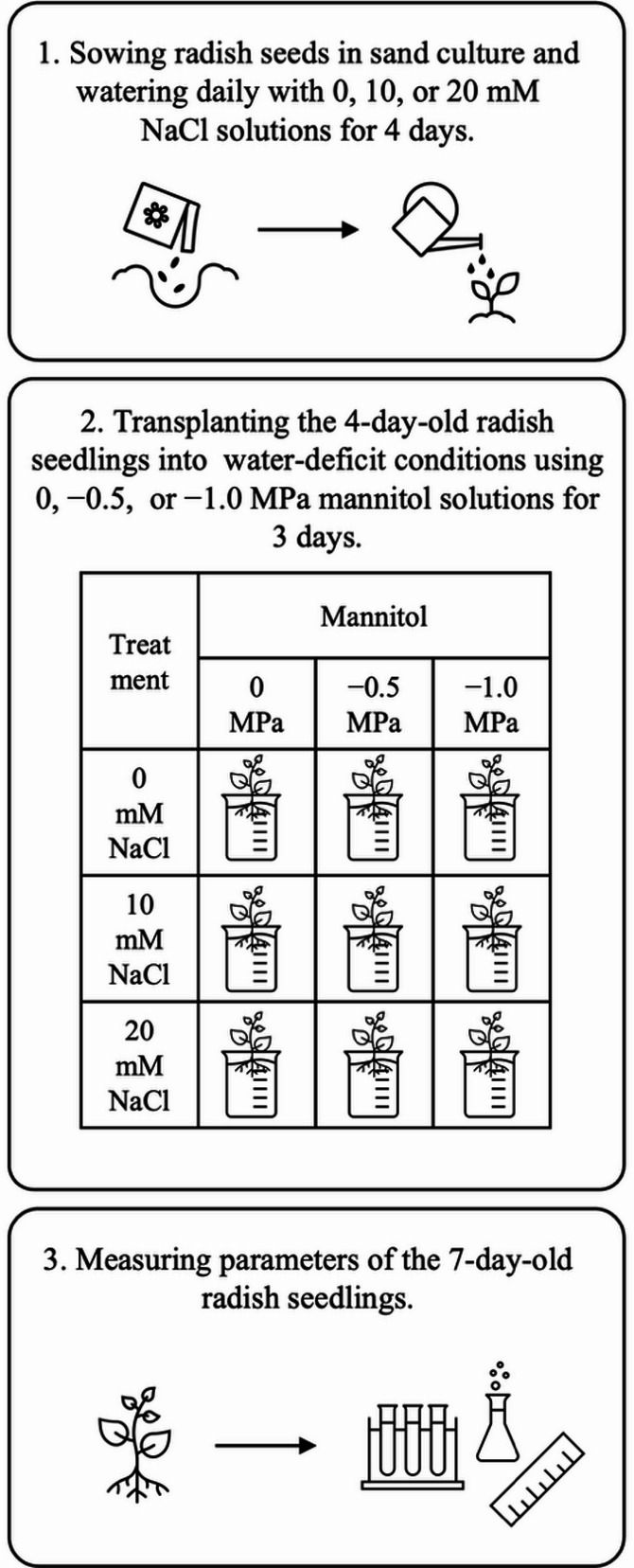
The design of experiment

Seeds of Chinese white radish (*Raphanus sativus* var. *longipinnatus* L.H. Bailey is a synonym of *Raphanus raphanistrum* subsp. *sativus* [powo.science.kew.org]) were purchased from Jia Tai Company (Bangkok, Thailand). The seeds were soaked in distilled water for 18 h prior to use. Ten fully imbibed seeds were selected and germinated in each plastic containers filled with 200 g of fine sand. The sand cultures were watered with 40 mL of NaCl solutions at 0 (control), 10, or 20 mM and an additional 10 mL of the corresponding solution was applied daily to each treatment. The seedlings were grown in a greenhouse under a 12 h light/12 h dark photoperiod at 30 ± 5 °C, 70 ± 5% relative humidity and a photosynthetic photon flux density of 3000 ± 100 lx. Four-day-old seedlings (the optimal time period for inducing maximal seedling growth [21]) from both the control and NaCl pretreatment groups were transferred to mannitol solutions without NaCl at 0 (control), − 0.5, or − 1.0 MPa for 3 days to induce water deficit. A factorial experiment was conducted using a completely randomized design (CRD). Each treatment included three biological replicates (*n* = 3), with replication for each measured parameter. The experimental design is illustrated in Fig. 1. After treatment, growth, yield, water status, chlorophyll and proline contents were measured in 7-day-old seedlings. Based on these results, the optimal NaCl pretreatment (10 mM) and water-deficit conditions (–0.5 MPa) were selected for further experiments. Under these conditions, photosynthetic and respiratory activities, sugar contents, and adenylate levels were subsequently analysed.

### Analysis and measurement

#### Seedling growth and yield parameters

Shoot height and root length were measured with a ruler and recorded in centimeters. Whole-seedling fresh weight (FW) was determined using a precision balance (Ohaus, USA). The seedlings were then dried at 80 °C for 2 days in a hot-air oven (Binder, USA) to obtain dry weight (DW). For yield assessment, the roots were removed from each fresh seedling and the remaining hypocotyls and cotyledons in each container were weighed to determine yield (g_FW_/seedling).

### Water status parameters

The FW of the whole seedlings was measured, followed by the turgid weight (TW) after soaking the samples in distilled water for 4 h until saturation. The DW was determined by drying the whole seedlings at 80 °C, as previously described. The relative water content (RWC) was calculated according to the modified method of Smart and Bingham [22]: RWC (%) = [(FW–DW)/(TW–DW)]×100. The water content (WC) and degree of succulence (DS) were calculated following the modified method of Zeng et al. [23]: WC (g/g_DW_) = (FW–DW)/DW and DS = FW/DW.

### Shoot chlorophyll contents

The chlorophyll content was measured according to the method of Chappelle et al. [24]. Fresh shoot samples, including the cotyledons and hypocotyl, were soaked in dimethyl sulphoxide (DMSO) for 48 h. The absorbance of the solution at 648 nm and 664 nm was measured using a spectrophotometer. Chlorophyll a (Chl a) and chlorophyll b (Chl b) contents were calculated as: Chl a (µg/g_FW_) = (12.25×A664)–(2.79×A648) and Chl b (µg/g_FW_) = (21.50×A648)–(5.10×A664). These values were recalculated in µg/g_DW_ and the chlorophyll a/b ratio was determined.

### Proline content

The proline content was determined following the methods described by Bates et al. [25] and Ghoulam et al. [26]. Fresh samples of the whole seedlings were ground in 80% methanol, filtered and treated with an acid ninhydrin reagent. The mixture was incubated at 100 °C, then placed on ice to stop the reaction, followed by the addition of toluene. The absorbance of the supernatant was measured at 528 nm using a spectrophotometer (BMG LABTECH, Germany). Proline content was calculated using a standard curve, expressed in µmol/g_FW_ and later recalculated in µmol/g_DW_.

### Photosynthetic activity (Hill’s reaction)

The Hill’s reaction assay was performed following the modified methods of Dean and Miskiewicz [27] and Latkowska et al. [28]. Fresh shoot samples were homogenised in the grinding medium (0.04 M K₃PO₄, 0.008 M KCl and 0.04 M sucrose; pH 6.8), filtered and centrifuged at 4000 rpm for 5 min at 0 °C. The pellet was then resuspended in the same medium to obtain the chloroplast suspension and mixed with 0.04 M phosphate buffer (pH 6.8), 0.1% 2,6-dichlorophenolindophenol (DCPIP) and distilled water. The absorbance at 600 nm was recorded as A_600s_ using a spectrophotometer, with the chloroplast suspension lacking DCPIP as the blank. The mixture was illuminated with a 100 W light bulb positioned 15 cm above the sample for 10 min and the absorbance was measured again as A_600E_. The Hill’s reaction activity was calculated as: [(A_600S_−A_600E_)/A_600S_]×100 and expressed as the percentage change in absorbance.

### Respiratory activity (CO_2_ production)

Fresh seedling samples were placed in sealed tubes for 1 h and the CO₂ concentrations in the headspaces were measured using a Bridge Analyzer (Bridge Analyzer Inc., USA). The production of CO_2_ was calculated as: Production of CO_2_ = [(%CO_2_/100)×d×chamber volume]/(sample fresh weight×time), where d represents the CO₂ conversion factor (1.795 kg/m^3^ at 25 °C) and 1 atm is defined as the mass of CO₂ (mg) contained in 1 L of air at 1% CO₂ by volume and expressed as µg/g_FW_·h.

### Sugar contents

The sugar content was measured using a modified method based on Kamata and Uemura [29]. Fresh seedling samples were ground in distilled water, followed by centrifugation at 15,000 g for 20 min at room temperature. The resulting supernatant was collected and filtered through a 0.45 μm membrane filter before analysis by high-performance liquid chromatography (HPLC) (Agilent Corp., USA) performed with an Asahipak NH2P-50 4E column (Shodex, Japan) maintained at 30 °C and equipped with a refractive index detector. Sucrose, glucose and fructose were used as standards. Elution was carried out using a mixture of deionised water and acetonitrile (25:75 v/v) at a flow rate of 1.0 mL/min. Each sugar content was initially expressed in mg/g_FW_ and later converted to mg/g_DW_. Sample HPLC chromatograms of standard sugar contents are presented in Supplementary Fig. A.

### Adenylate contents

The adenosine triphosphate (ATP), adenosine diphosphate (ADP) and adenosine monophosphate (AMP) contents were measured using a modified method based on Liu et al. [30]. Fresh seedling samples were ground in 0.6 M perchloric acid, followed by centrifugation at 6,000 g for 10 min at 4 °C. The resulting supernatant was collected and the pH was adjusted to 6.5–6.8 by adding 1 M KOH and then filtered through a 0.45 μm membrane before analysis by HPLC, with UV absorbance at 254 nm. The HPLC analysis was performed with an Eclipse XDB-C18 column (4.6×150 mm, Agilent Corp., USA), with gradient elution using 50 mM potassium phosphate buffer (pH 7.0) and 100% acetonitrile at a flow rate of 1.2 mL/min. ATP, ADP and AMP were used as standards. The amount of adenosine phosphate was calculated using a standard curve in µg/g_FW_ and converted to µg/g_DW_. The ratios of ATP/ADP and ATP/AMP were also determined. The total adenylate pool (TAP) and the adenylate energy charge (AEC) were calculated as: TAP = ATP+ADP+AMP and AEC = (ATP+0.5ADP)/TAP. Sample HPLC chromatograms of standard adenylate contents are presented in Supplementary Fig. B.

### Statistical analysis

Data from each experiment were statistically analysed using RStudio (version 2025.05.0 + 496). Two-way ANOVA was performed, followed by the calculation of mean values ± standard deviation and pairwise comparisons using Duncan’s multiple range test (*p* < 0.05) implemented in the R package ‘agricolae’ (version 1.3-5).

## Results

### Growth parameters

**Fig. 2:**
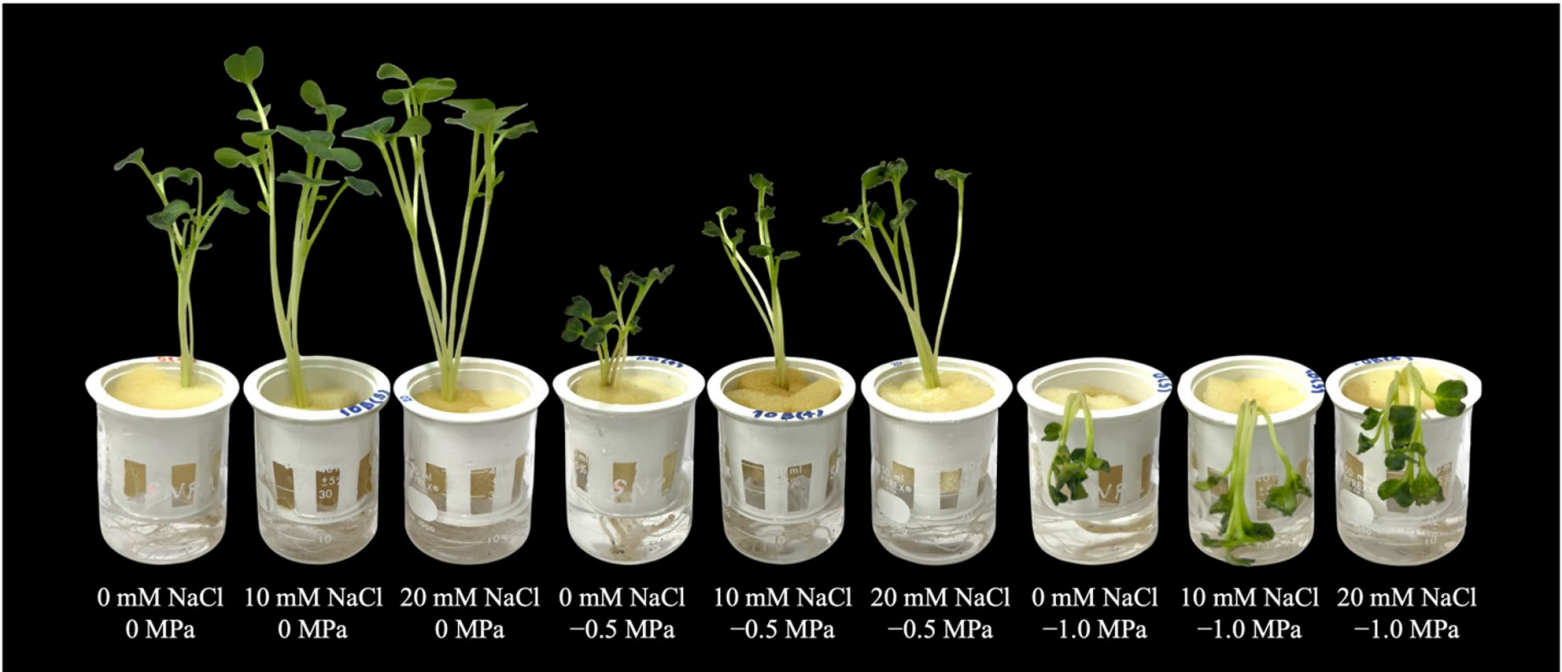
Effects of 0, 10 and 20 mM NaCl pretreatments on growth of 7-day-old Chinese white radish seedlings under 0, − 0.5 and − 1.0 MPa mannitol solutions

**Fig. 3:**
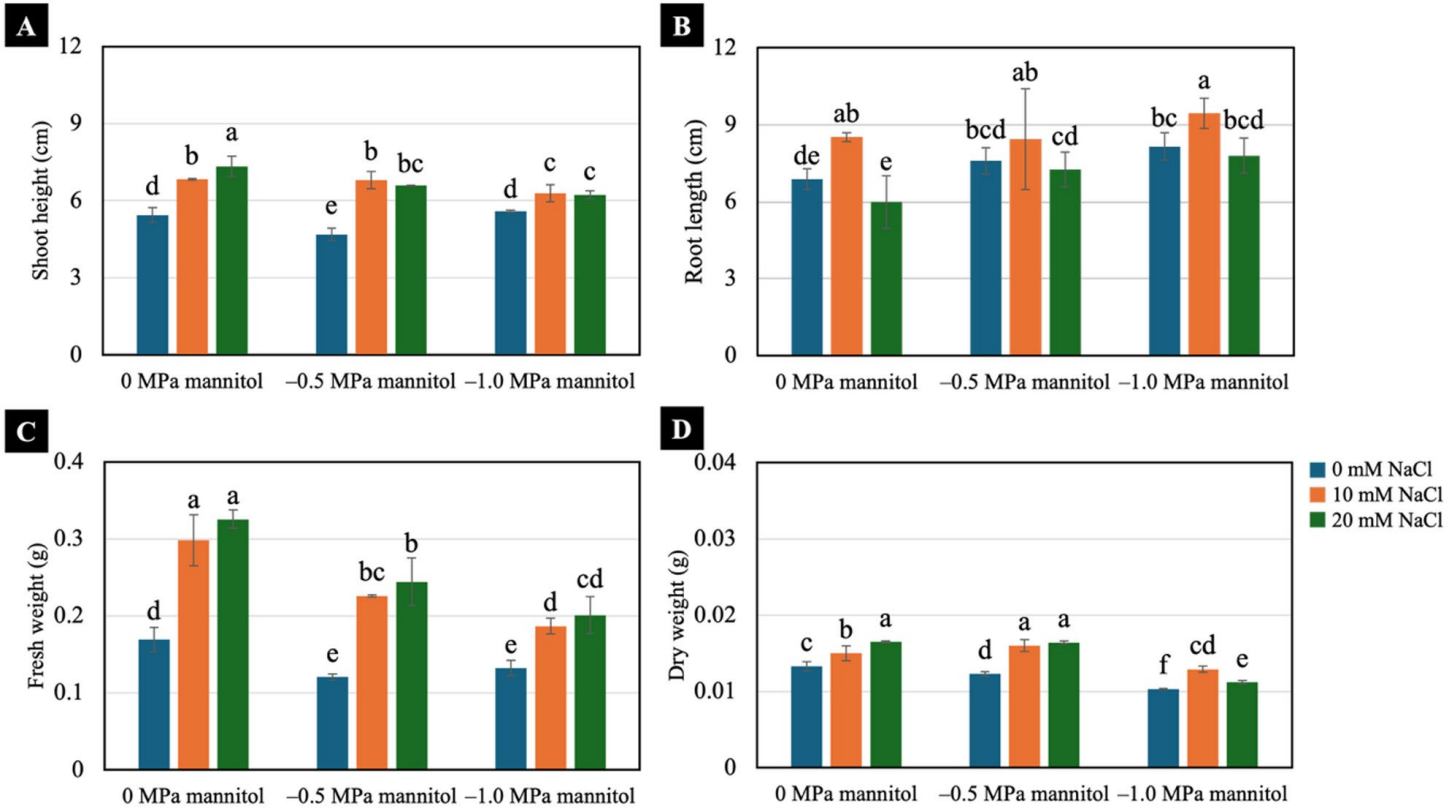
Effects of low NaCl pretreatments on shoot height (**A**), root length (**B**), fresh weight (**C**) and dry weight (**D**) of 7-day-old Chinese white radish seedlings under water-deficit conditions. The results are the means of three replicates, with the standard deviation (SD) indicated by vertical bars. Different letters (a-f) of the same parameter were considered significantly different at *p* < 0.05 by Duncan’s multiple range tests

Increasing water-deficit severity reduced growth of 7-day-old Chinese white radish seedlings (Fig. 2). Under well-watered conditions (0 MPa), seedlings pretreated with 10 mM and 20 mM NaCl showed significantly greater shoot height and fresh weight than non-treated seedlings. These pretreatments also maintained higher values under water-deficit conditions (–0.5 and − 1.0 MPa) compared with non-pretreated seedlings (Fig. 3A and C). Pretreatment with 10 mM NaCl resulted in longer root length under both well-watered and water-deficit conditions compared with all the other treatments (Fig. 3B). Seedlings pretreated with 10 mM and 20 mM NaCl exhibited higher dry weight than non-treated seedlings under both well-watered and water-deficit conditions, while exposure to − 1.0 MPa water deficit significantly reduced dry weight across all the treatments (Fig. 3D).

### Yield and water-status parameters

**Fig. 4:**
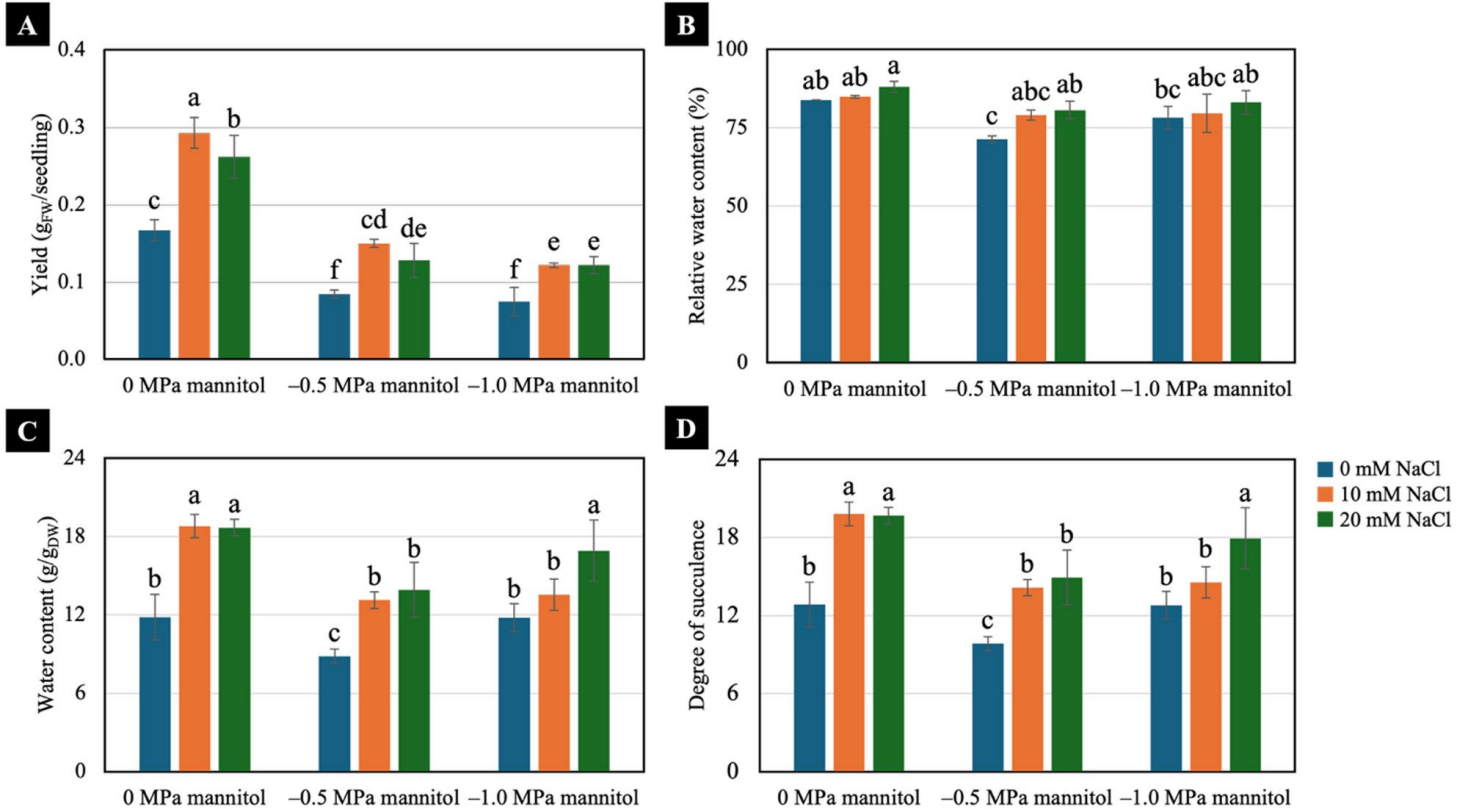
Effects of low NaCl pretreatments on yield (**A**), relative water content (**B**), water content (**C**) and degree of succulence (**D**) of 7-day-old Chinese white radish seedlings under water-deficit conditions. The results are the means of three replicates, with the standard deviation (SD) indicated by vertical bars. Different letters (a-f) of the same parameter were considered significantly different at *p* < 0.05 by Duncan’s multiple range tests

Increasing water-deficit severity reduced the yield of 7-day-old Chinese white radish seedlings, while NaCl-pretreated seedlings exhibited higher yields than non-treated seedlings under both well-watered and water-deficit conditions. Exposure to − 1.0 MPa water-deficit level had the most severe effect compared with the other treatments (Figs. 2 and 4A). In term of water status, no significant differences in relative water content (RWC) were observed between the NaCl-pretreated and non-pretreated seedlings both under well-watered (0 MPa) and − 1.0 MPa water-deficit conditions. However, seedlings pretreated with the NaCl exhibited higher RWC than non-treated seedlings under − 0.5 MPa water deficit (Fig. 4B), while 10 and 20 mM NaCl pretreatment significantly induced higher water content and degree of succulence under well-watered and water-deficit conditions (Fig. 4C and D).

### Physiological parameters

**Fig. 5:**
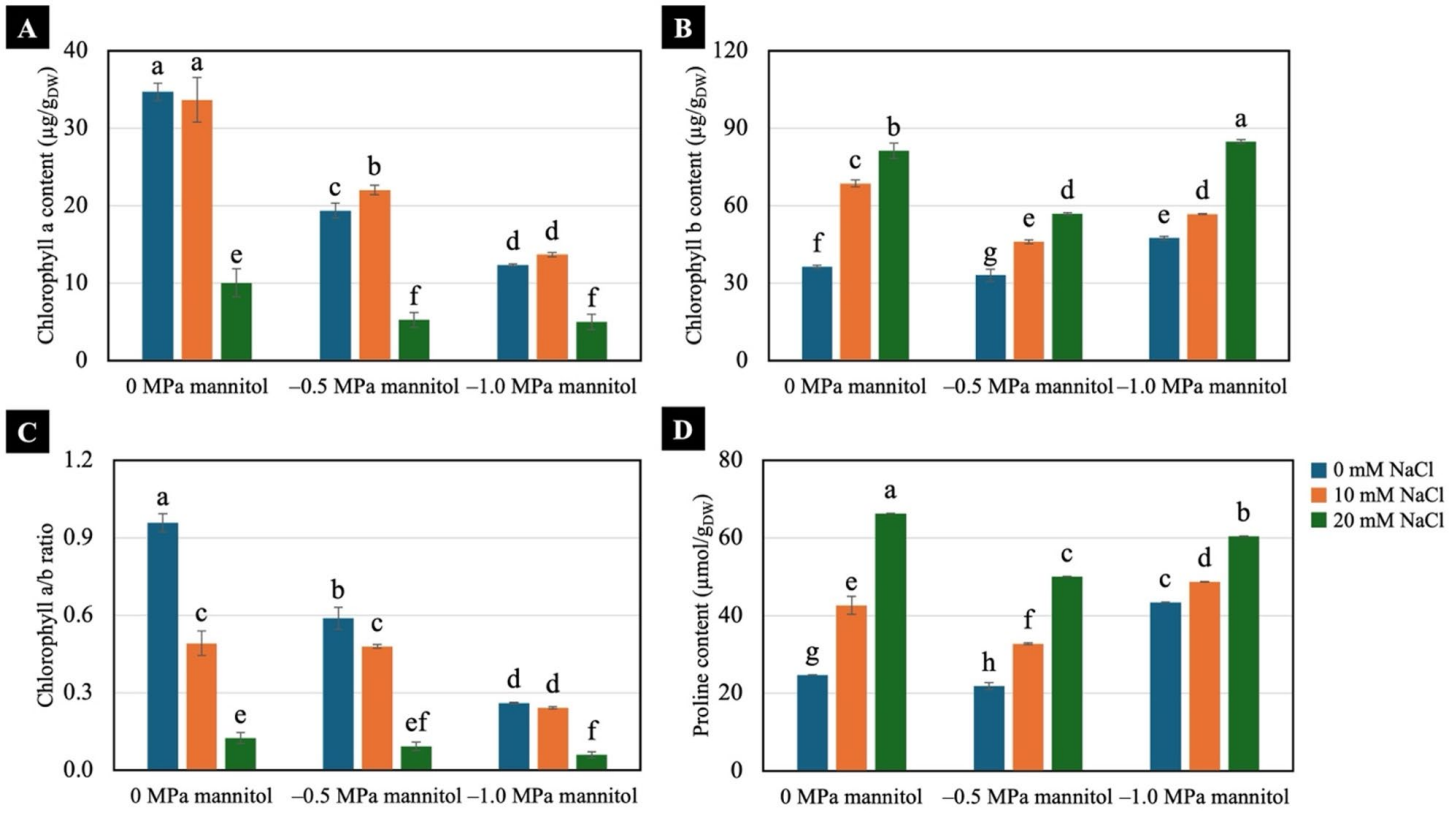
Effects of low NaCl pretreatments on chlorophyll a content (**A**), chlorophyll b content (**B**), chlorophyll a/b ratio (**C**) and proline content (**D**) of 7-day-old Chinese white radish seedlings under water-deficit conditions. The results are the means of three replicates, with the standard deviation (SD) indicated by vertical bars. Different letters (a-h) of the same parameter were considered significantly different at *p* < 0.05 by Duncan’s multiple range tests

Increasing water-deficit levels reduced chlorophyll a content in 7-day-old Chinese white radish seedlings. However, seedlings pretreated with 10 mM NaCl maintained higher chlorophyll a level than those pretreated with 20 mM NaCl under both well-watered and water-deficit conditions (Fig. 5A). By contrast, chlorophyll b content increased in response to NaCl pretreatment under both conditions (Fig. 5B). As a result, the chlorophyll a/b ratio decreased in seedlings subjected to NaCl pretreatment, with 20 mM NaCl causing the highest decline under both well-watered and water-deficit conditions (Fig. 5C). Proline content increased in response to NaCl pretreatment and water-deficit conditions, with 20 mM NaCl inducing higher accumulation than 10 mM NaCl compared with the non-treated seedlings (Fig. 5D).

### Main and interaction effects of NaCl pretreatment and water deficit on the measured parameters

The interaction between NaCl pretreatment and water-deficit condition significantly affected CO₂ production, sugar contents, and energy status parameters. Highly significant NaCl×water deficit interactions were detected for most variables, including CO₂ production, fructose, glucose, sucrose, ATP, ADP, AMP, total adenylate pool (TAP), ATP/ADP and ATP/AMP ratios, and adenylate energy charge (AEC) (*p* < 0.001–0.01). In contrast, Hill’s reaction activity and sucrose content were not influenced by the main effect of NaCl pretreatment. Meanwhile, CO₂ production exhibited no significant main effect of water-deficit condition (Table 1).

**Table 1:** Interaction effects and main effects of NaCl pretreatment and water deficit on hill’s reaction activity, production of CO_2_, sugar contents and energy status of 7-day-old Chinese white radish seedlings

Parameters	NaCl pretreatment	Water deficit	NaCl×Water deficit
Hill’s reaction activity	ns	***	ns
Production of CO_2_	***	ns	***
Fructose content	***	***	***
Glucose content	*	***	***
Sucrose content	ns	***	**
ATP content	***	***	***
ADP content	***	**	***
AMP content	***	***	***
TAP	***	***	***
ATP/ADP	***	***	***
ATP/AMP	***	***	***
AEC	***	***	***

*** *p* < 0.001, ** 0.001 ≤ *p* < 0.01, * 0.01 ≤ *p* < 0.05, ns *p* ≥ 0.05

### Hill’s reaction activity and production of CO_2_

**Fig. 6:**
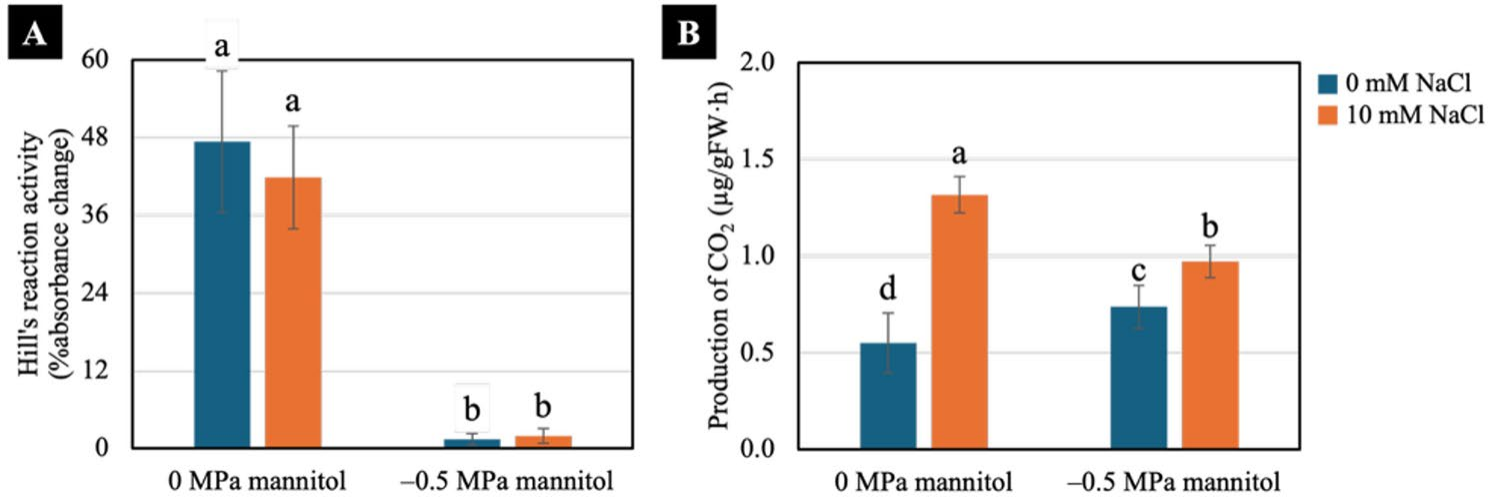
Effects of water deficit on Hill’s reaction activity (**A**) and production of CO_2_ (**B**) of 7-day-old Chinese white radish seedlings pretreated with NaCl. The results are the means of three replicates, with the standard deviation (SD) indicated by vertical bars. Different letters (a-d) of the same parameter were considered significantly different at *p* < 0.05 by Duncan’s multiple range tests

Water deficit significantly reduced Hill’s reaction activity in both non-pretreated and NaCl-pretreated seedlings compared with well-watered condition (Fig. 6A). By contrast, NaCl pretreatment significantly increased production of CO_2_ in both well-watered and water-deficit groups, whereas water deficit significantly increased production of CO_2_ in non-treated seedlings compared with the control under well-watered condition (Fig. 6B).

### Sugar contents

**Fig. 7:**
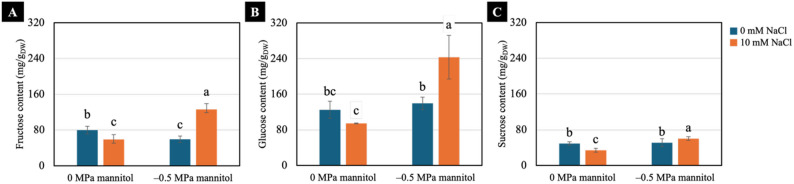
Effects of water deficit on fructose content (**A**), glucose content (**B**) and sucrose content (**C**) of 7-day-old Chinese white radish seedlings pretreated with NaCl. The results are the means of three replicates, with the standard deviation (SD) indicated by vertical bars. Different letters (a-c) of the same parameter were considered significantly different at *p* < 0.05 by Duncan’s multiple range tests

Both 10 mM NaCl pretreatment and the − 0.5 MPa water-deficit condition significantly increased the accumulation of fructose, glucose, and sucrose compared with all other treatments. Conversely, under well-watered condition (0 MPa), seedlings pretreated with 10 mM NaCl showed the lowest sugar levels among treatments (Fig. 7).

### Adenylate contents

**Fig. 8:**
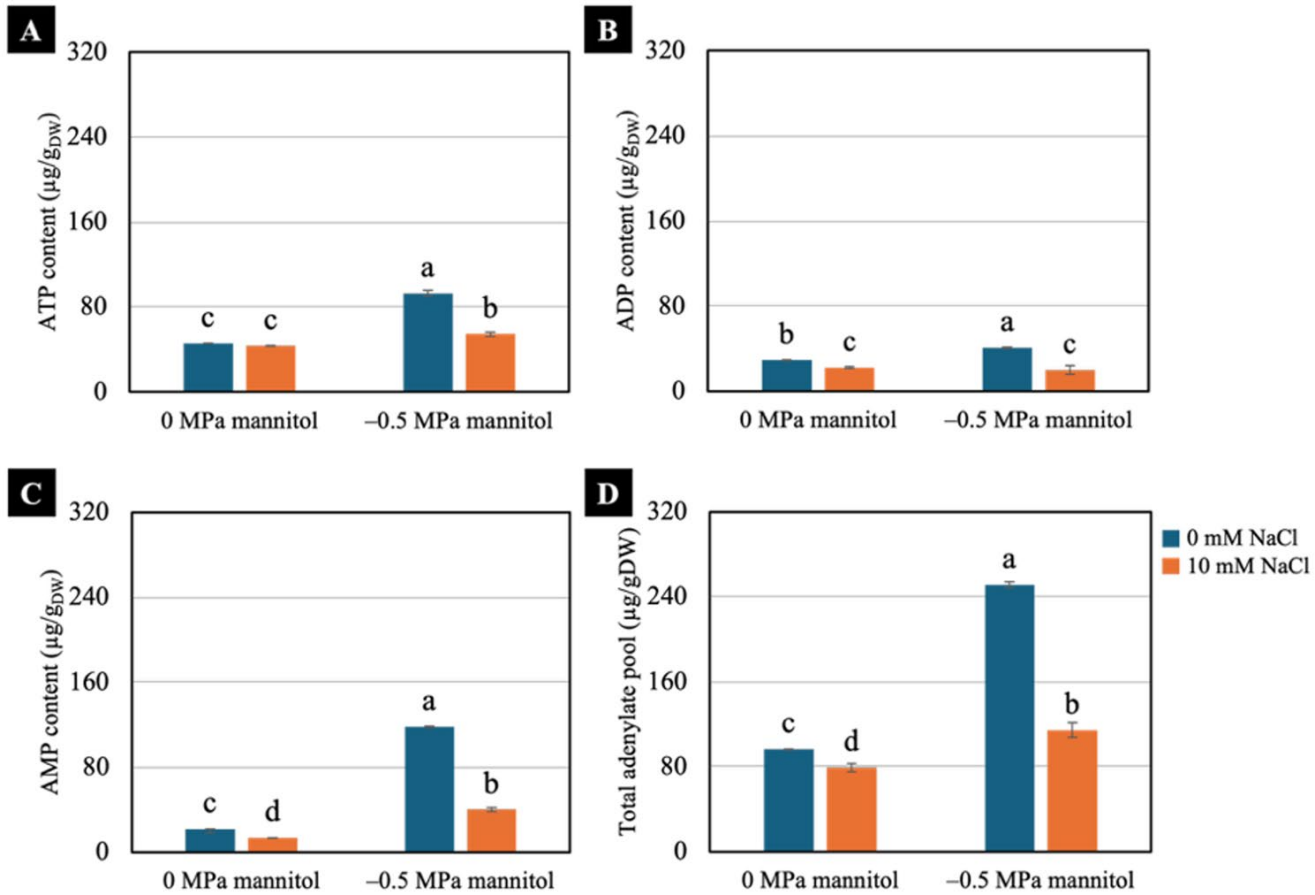
Effects of water deficit on adenosine triphosphate (ATP) content (**A**), adenosine diphosphate (ADP) content (**B**), adenosine monophosphate (AMP) content (**C**) and total adenylate pool (TAP) (**D**) of 7-day-old Chinese white radish seedlings pretreated with NaCl. The results are the means of three replicates, with the standard deviation (SD) indicated by vertical bars. Different letters (a-d) of the same parameter were considered significantly different at *p* < 0.05 by Duncan’s multiple range tests

Water deficit significantly increased contents of ATP, ADP, AMP, and the total adenylate pool in non-pretreated seedlings. Under water-deficit condition, seedlings pretreated with 10 mM NaCl also exhibited higher ATP, AMP, and total adenylate levels compared with non-pretreated seedlings under well-watered condition. In contrast, under well-watered condition (0 MPa), 10 mM NaCl-pretreated seedlings showed the lowest levels of these adenylates among all treatments (Fig. 8).

### Adenylate ratios and energy charge

**Fig. 9:**
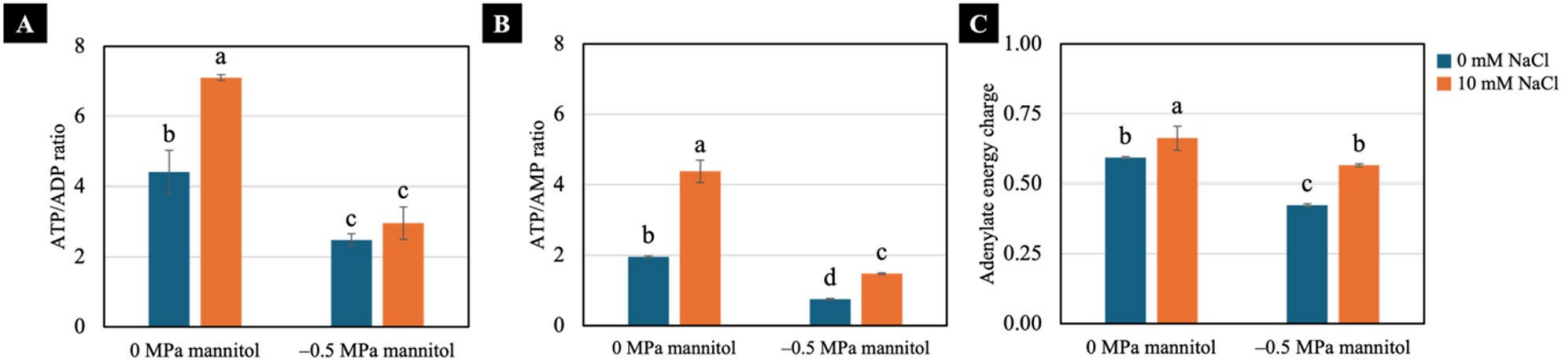
Effects of water deficit on ATP/ADP ratio (**A**), ATP/AMP ratio (**B**) and adenylate energy charge (**C**) of 7-day-old Chinese white radish seedlings pretreated with NaCl. The results are the means of three replicates, with the standard deviation (SD) indicated by vertical bars. Different letters (a-d) of the same parameter were considered significantly different at *p* < 0.05 by Duncan’s multiple range tests

Well-watered condition significantly increased the ratios of ATP/ADP, ATP/AMP, and the adenylate energy charge in 10 mM NaCl pretreated seedlings. Under water-deficit condition, seedlings pretreated with 10 mM NaCl also exhibited higher values of these parameters compared with non-pretreated seedlings, which showed the lowest levels among all treatments (Fig. 9).

## Discussion

### Growth, yield and water status

Water deficit induced by − 0.5 and − 1.0 MPa mannitol significantly reduced the growth and yield of 7-day-old Chinese white radish seedlings (Figs. 2, 3 and 4A). In contrast, NaCl pretreatment supported overall improvements in these parameters under water-deficit conditions. Consistent with the observed growth and yield responses, NaCl pretreatment also better maintained seedling water status, as indicated by higher water content and degree of succulence during water deficit (Fig. 4C and D). Collectively, these results suggest that low-level NaCl pretreatment enhances water retention and promotes more stable growth under water-deficit conditions. Previous studies have likewise demonstrated growth-promoting effects of low NaCl concentrations in various plant species, primarily through enhanced water uptake and osmotic regulation. For example, Hongqiao et al. [10] reported that treatment with 5 mM NaCl enhanced biomass production and nutrient accumulation in *Arabidopsis thaliana* seedlings, whereas Jēkabsone et al. [31] demonstrated that 20–100 mM NaCl improved water content and growth in juvenile *Mesembryanthemum crystallinum*. Similarly, Masepan et al. [21] showed that 10–20 mM NaCl stimulated cotyledon expansion in Chinese white radish seedlings through aquaporin-mediated water transport. Moreover, low-NaCl pretreatment has been reported to induce tolerance to water-deficit stress in several plant species [12–14].

### Photosynthetic pigments

Both water deficit and NaCl pretreatment influenced chlorophyll levels, but the responses differed among pigment types (Fig. 5A and B). Chlorophyll a is the primary pigment for light harvesting and electron excitation and a decline in the chlorophyll a/b ratio is often interpreted as a marker of photosynthetic sensitivity to water-deficit stress [32]. In our study, chlorophyll a and the chlorophyll a/b ratio decreased with increasing NaCl concentration and water deficit (Fig. 5A and C), consistent with previous reports showing that water deficit reduced photosynthetic pigments in radish and that chlorophyll a declined more sharply than chlorophyll b [33]. Seedlings pretreated with 10 mM NaCl maintained chlorophyll a content and the a/b ratio at levels comparable to the untreated control, whereas 20 mM NaCl markedly reduced both parameters across all conditions. By contrast, chlorophyll b increased with higher NaCl pretreatment regardless of water-deficit level (Fig. 5B). Chlorophyll b is part of the light-harvesting antenna [34] and its increase may reflect an NaCl-induced adjustment in antenna composition rather than a direct water-deficit response. Overall, these results indicated that 10 mM NaCl was more effective at maintaining chlorophyll composition under water-deficit conditions, while 20 mM NaCl was above the optimal range for preserving pigment stability.

### Proline content

Proline functions as a compatible solute, stabilising membranes and proteins, scavenging ROS and facilitating osmotic adjustment. It also serves as a useful indicator of abiotic stress, as drought and salt stress are well known to induce pronounced proline accumulation [35, 36]. In our study, proline content increased with water deficit in non-treated seedlings and in those pretreated with 10 mM NaCl (Fig. 5D), indicating a water deficit induced osmoprotective response. By contrast, seedlings receiving 20 mM NaCl accumulated high proline even under well-watered condition and showed declined rather than increased proline levels under severe water deficit, contrasting with the normal water-deficit-induced pattern observed at 0 and 10 mM NaCl (Fig. 5D). The early induction of proline at 20 mM suggested that this NaCl level was too high to function as an effective priming treatment, triggering a strong response even before water deficit occurred. By comparison, 10 mM NaCl allowed the seedlings to accumulate proline progressively with increasing water-deficit levels, without causing excessive baseline levels, which possibly contributed to the higher water retention observed in NaCl-pretreated seedlings in the previous section. This finding aligned with previous reports indicating that NaCl pretreatment activated osmolyte biosynthesis as a preparatory response to subsequent stress [21, 35, 37]. Thus, 10 mM NaCl under − 0.5 MPa mannitol was selected further analyses, as this water-deficit level induced measurable negative effects while still allowing the seedlings to survive throughout the experimental period.

### Photosynthetic and respiratory activities

Under water deficit, both non-treated and NaCl-pretreated seedlings showed a sharp decline in Hill’s reaction activity (Fig. 6A). This indicated that water deficit strongly suppressed the light-dependent reactions of photosynthesis regardless of NaCl pretreatment, with only water-deficit condition having a significant effect on Hill’s reaction activity (Table 1). As a fundamental process driving primary metabolism, photosynthesis is essential for supporting plant performance under drought stress [38]. Under water deficit, reduced CO₂ diffusion caused by stomatal closure, together with ROS overproduction which impairs the photosynthetic electron transport chain [39] and consequently disrupts ATP synthesis [40], particularly in the non-treated seedlings. However, additional indicators, such as measurements of specific ROS, are still required to fully address this point.

Additional to the reduction in photosynthesis, the increase in respiration observed in the non-treated seedlings under water deficit was consistent with the expected shift toward higher respiratory carbon loss when photosynthetic carbon gain was restricted [41]. This was reflected in their elevated CO₂ production compared with non-treated seedlings under well-watered conditions (Fig. 6B). However, both NaCl-pretreated groups exhibited a substantially greater rise in CO₂ release than their respective controls (Fig. 6B), indicating that NaCl pretreatment enhanced respiratory activity independently of water deficit (Table 1). This suggested that NaCl pretreatment may elevate basal metabolic activity or accelerate carbon turnover, rather than acting solely as a drought-induced compensatory response. Taken together, these results indicated that NaCl pretreatment did not mitigate the inhibition of light-dependent reactions under water-deficit condition, but instead modulated downstream metabolic processes, particularly those associated with sugar mobilisation, osmotic adjustment and cellular energy balance. These aspects are examined and duscussed in the following section.

### Sugar contents

Soluble sugars such as fructose and glucose act as readily available osmolytes and energy sources that help cells maintain hydration and basic metabolic activity during water-deficit stress [42–44]. Sucrose also contributes to osmotic protection, while serving as a more stable carbon reserve that supports continued stress tolerance [45, 46]. In our study, neither NaCl pretreatment alone nor water deficit alone resulted in substantial alterations in sugar levels compared with the non-treated, well-watered seedlings (Fig. 7), indicating that neither treatment increased sugar levels by itself. However, seedlings pretreated with NaCl and subsequently exposed to water deficit showed a marked increase in fructose, glucose and sucrose compared with other conditions. This contrasting response reflected a priming effect, in which previous exposure to mild salinity enhanced the plant’s ability to activate sugar accumulation when water deficit occurred [47, 48]. The coordinated increase in these soluble sugars contributed to maintaining cellular homeostasis under water deficit by supporting osmotic balance and essential metabolic processes [6, 44, 46].

### Energy status

Water deficit induces substantial energy consumption in non-NaCl-pretreated seedlings, as reflected by the marked reduction in the ATP/ADP ratio and by ATP/AMP values (Fig. 9A and B) that were sufficiently low to indicate a potential cellular energy deficit [6]. Such energy limitation is expected to activate SnRK1, the central energy sensor that promotes catabolic metabolism and suppresses energy-consuming biosynthetic pathways to restore cellular energy balance [7, 8]. Consistent with this regulatory shift, the non-pretreated seedlings exhibited pronounced increases in ATP, ADP and AMP, resulting in elevated TAP (Fig. 8) and enhanced CO₂ production under water-deficit condition when compared to the non-treated seedlings under well-watered condition (Fig. 6B). This increase in total adenylates therefore represents not an improvement in energy availability, but rather a compensatory response driven by SnRK1-mediated enhancement of respiratory carbon turnover to mitigate ATP depletion [41]. Despite this compensatory accumulation, the energetic demands imposed by water deficit exceeded the capacity for energy restoration. This was evident from the persistently low ATP/ADP and ATP/AMP ratios and the reduced adenylate energy charge (AEC) (Fig. 9), indicating that most adenylates were present in low-energy forms and thus insufficient to support metabolic needs. The resulting disruption of cellular energy homeostasis is consistent with the pronounced growth suppression observed in the non-NaCl-pretreated seedlings under water-deficit conditions.

By contrast, seedlings pretreated with 10 mM NaCl, under both well-watered and water-deficit conditions, showed increased CO₂ production (Fig. 6B), implying enhanced ATP synthesis. These seedlings maintained sufficient energy. Their cellular energy status remained stable, SnRK1 was not activated and ATP, ADP and AMP levels were maintained without excessive accumulation, thereby preserving the adenylate pool balance (Fig. 8). As a result, these seedlings sustained higher ATP/ADP and ATP/AMP ratios and a higher AEC, reflecting a more balanced energetic homeostasis. NaCl pretreatment thus mitigated the adverse effects of drought on energy metabolism, supporting efficient ATP regeneration and stabilising the relationship between ATP production and consumption, which contributed to improved stress tolerance.

**Fig. 10:**
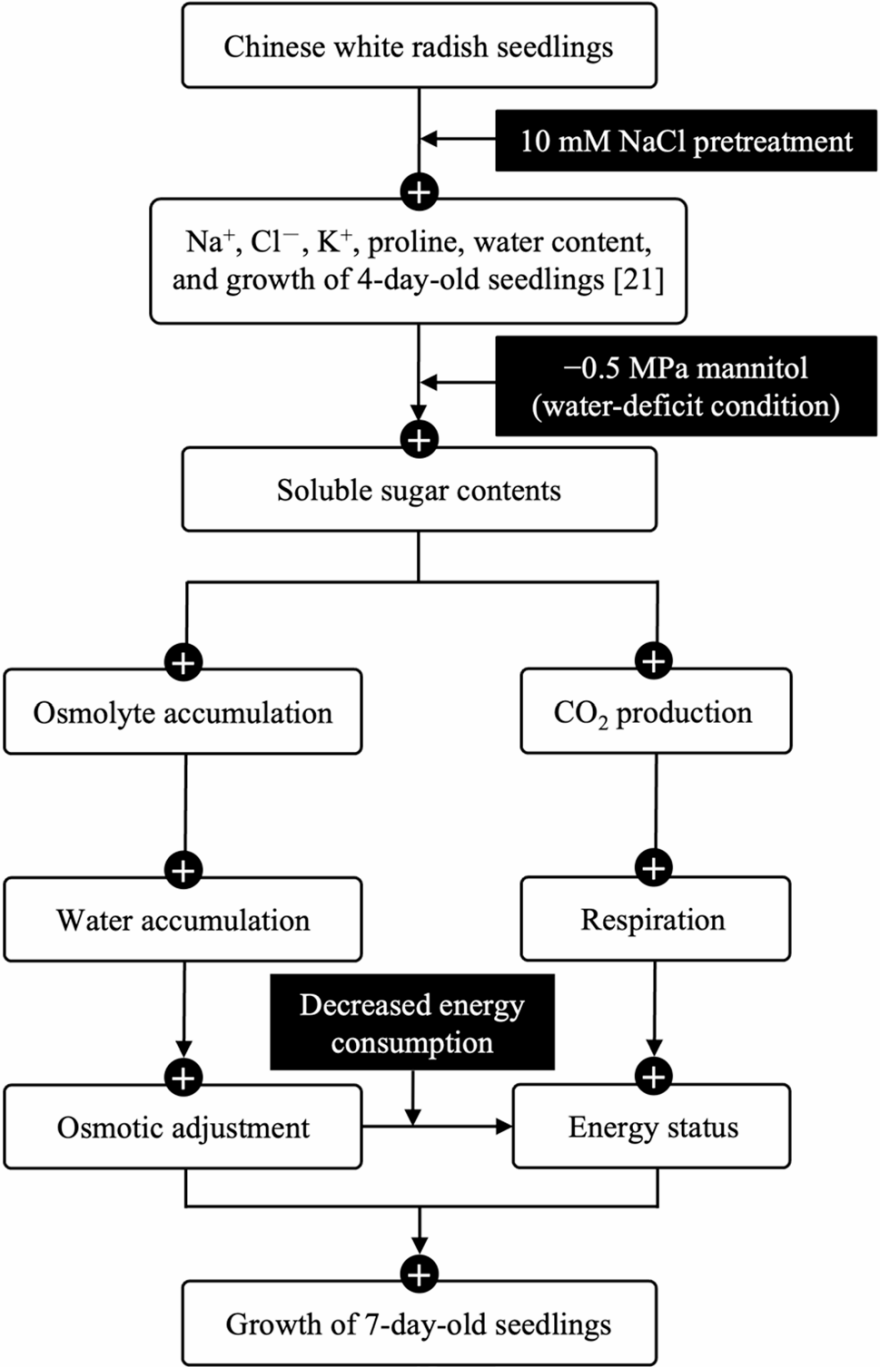
Schematic representation of how 10 mM NaCl pretreatment improve growth of 7-day-old Chinese white radish seedlings under water deficit condition

Our findings extend previous observations by providing new insights into how NaCl pretreatment enhances the growth of Chinese white radish seedlings under water-deficit conditions through osmotic adjustment and modulation of metabolic energy dynamics, as evidenced by favorable adenylate ratios and AEC. This integrative mechanism underscores the potential of NaCl priming as an effective strategy for improving water-deficit tolerance during early seedling development. The proposed mechanism underlying these findings is illustrated in Fig. 10.

Further studies are needed to determine whether the NaCl-induced tolerance observed at the sprout stage is maintained throughout later developmental phases, including root storage organ formation. Investigating the long-term effects on growth, energy metabolism, and yield will be important for evaluating the practical applicability of NaCl pretreatment in crop production. Moreover, exploring the molecular mechanisms underlying these responses, particularly the transcriptional regulation of genes involved in osmolyte biosynthesis, energy metabolism, and stress signaling, will provide deeper insights into how NaCl priming coordinates osmotic and energy-related pathways to enhance water deficit tolerance in Chinese white radish seedlings.

## Conclusions

In conclusion, NaCl pretreatment at 10- and 20-mM enhanced shoot height, fresh weight, yield, water content, degree of succulence, and proline accumulation in Chinese white radish seedlings subjected to water deficit induced by − 0.5 and − 1.0 MPa mannitol. Among the treatments, 10 mM NaCl pretreatment combined with a moderate water deficit (− 0.5 MPa) was the optimal condition, resulting in superior shoot performance, higher chlorophyll a content, and an increased chlorophyll a/b ratio. Further analysis revealed that seedlings pretreated with 10 mM NaCl exhibited increased CO₂ production, higher fructose, glucose, and sucrose contents, elevated ATP/ADP and ATP/AMP ratios, and a higher adenylate energy charge, collectively supporting improved seedling growth under − 0.5 MPa water-deficit condition.

## Supplementary Information

Supplementary Material 1.

## Data Availability

All data underlying the findings reported in this paper are available in the published article and its supplementary materials; further inquiries should be directed to the corresponding author upon reasonable request.
