# Supplementary material for: Low-NaCl pretreatment promotes seedling growth of Chinese white radish under water deficit via osmotic and energy regulation
Source: BMC Plant Biol. 2026 Jan 20;26:298. doi: 10.1186/s12870-026-08175-9 (PMC12903664; doi:10.1186/s12870-026-08175-9)
Supplement: Supplementary file 1 — Supplementary Material 1. [file 12870_2026_8175_MOESM1_ESM.docx]

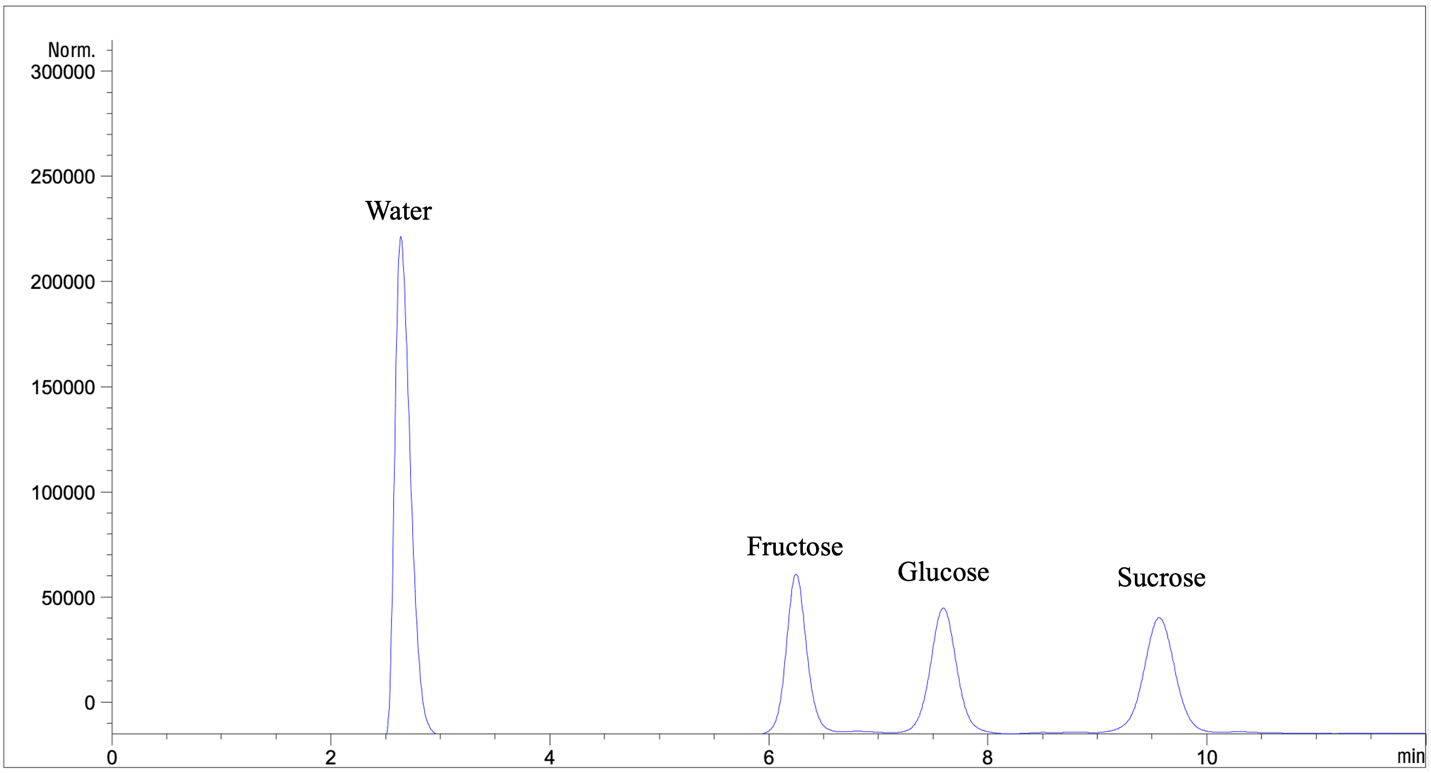


**Supplementary Fig. A** The HPLC chromatogram of 10 mg/mL sugars content.


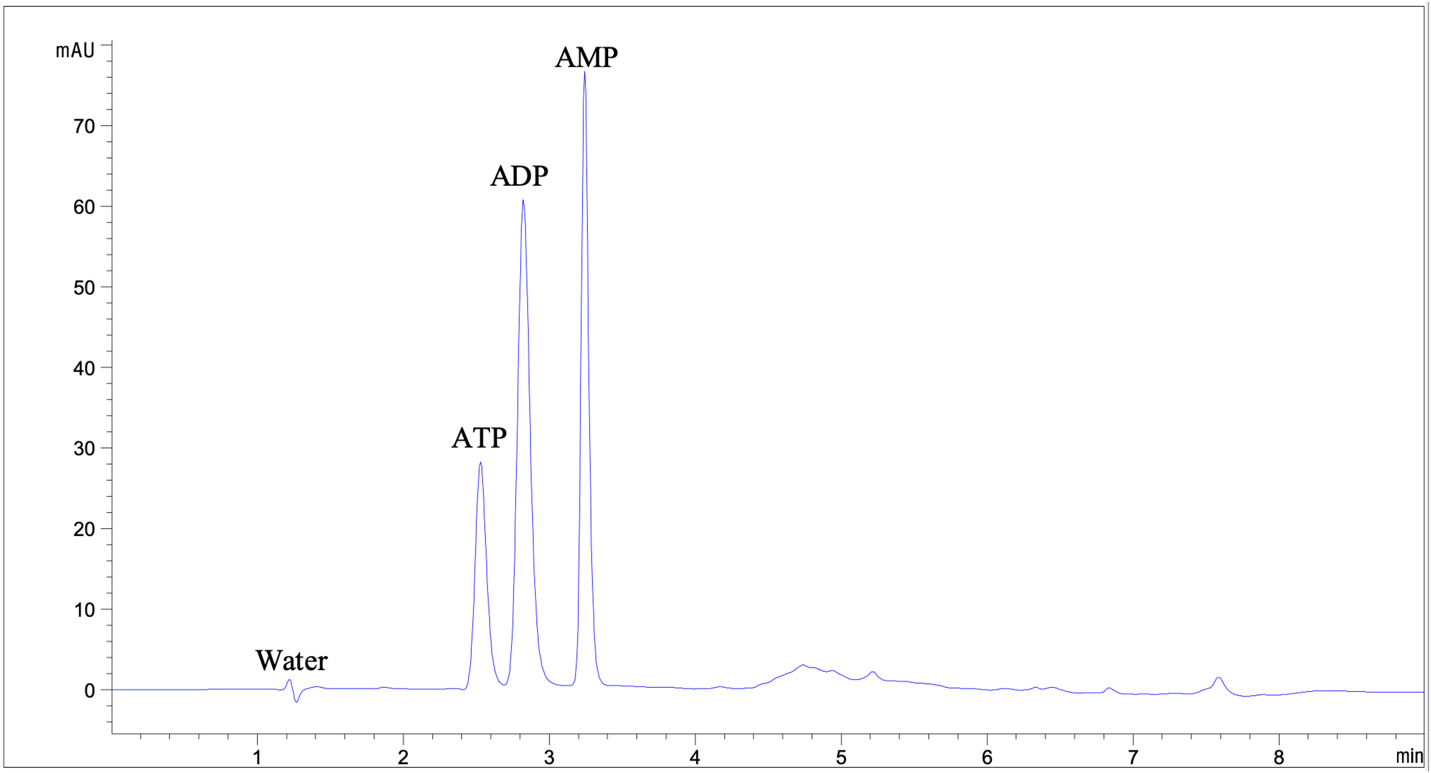


**Supplementary Fig. B** The HPLC chromatogram of 10 µg/mL adenylate content.
